# Predictive role of the neutrophil: lymphocyte ratio in acute kidney injury associated with off-pump coronary artery bypass grafting

**DOI:** 10.3389/fsurg.2022.1047050

**Published:** 2022-11-08

**Authors:** Ruiming Yu, Han Song, Yanwen Bi, Xiangbin Meng

**Affiliations:** Department of Cardiovascular Surgery, Qilu Hospital of Shandong University, Jinan, China

**Keywords:** coronary artery bypass grafting, acute kidney injury, risk factors, predictive model, cardiopulmonary bypass

## Abstract

**Objectives:**

This study aims to investigate whether the ratios of cell types in peripheral blood could be used as reliable predictors of off-pump coronary artery bypass grafting (CABG)-associated acute kidney injury (AKI).

**Materials and methods:**

We retrospectively reviewed patients (*n* = 420) undergoing off-pump CABG from January 1, 2021 to January 1, 2022 in Qilu Hospital of Shandong University. We used logistic regression analysis to identify the potential predictors of off-pump CABG-associated AKI and construct a predictive model. Receiver operating characteristic (ROC) curve analysis was used to evaluate the predictive ability of predictors and prediction models.

**Results:**

The prevalence of AKI associated with off-pump CABG was 20.95%. Patients in the AKI group had significantly higher ratios of peripheral blood cells on postoperative day (POD)1 than patients in the non-AKI group (*P* < 0.01). The area under the ROC curve (AUC) of the neutrophil:lymphocyte ratio (NLR) on POD1 for predicting off-pump CABG-associated AKI was 0.780 and the cutoff value was 20.07. Patients with high NLR on POD1 had a poor short-term prognosis. The AUC of the predictive model constructed by logistic regression analysis was 0.882. The sensitivity was 68.2% and the specificity was 93.1%.

**Conclusion:**

The NLR on POD1 was a reliable predictive biomarker of off-pump CABG-associated AKI. And we successfully construct a prediction model, which contribute to the early recognition and management of off-pump CABG-associated AKI.

## Introduction

Acute kidney injury (AKI) involves a sudden decline in renal function. Approximately 20% of adult patients develop AKI during hospitalization, 10% of whom require dialysis (1). Studies have indicated that even mild AKI is associated with a significantly increased risk of death, and the mortality in patients requiring renal replacement therapy (RRT) is 50%, which poses a huge challenge for medical professionals (1–4).

AKI development is heterogenous, and several mechanisms may be involved (5). Patients undergoing cardiac surgery are more likely to develop AKI due to hemodynamic changes, an increased inflammatory response, and use of nephrotoxic medications (6). Moreover, cardiac surgery-associated AKI (CSA-AKI) is associated independently with short-term and long-term mortality (7–9). Considering the high prevalence (≤42%) and severe effects of CSA-AKI, early recognition and intervention are very important (10).

The inflammatory cascade is considered to be a major event that aggravates injury to tubular epithelial cells and reduces the glomerular filtration rate (GFR) during the extension phase: this represents the most promising phase for successful treatment and intervention of AKI (11). Therefore, the predictive role of inflammatory response-related biomarkers in CSA-AKI has been studied extensively.

The relevant ratios of different cell types in peripheral blood are able to reflect the inflammation and have been found to be potential predictors of AKI after acute type-A aortic dissection, on-pump coronary artery bypass grafting (CABG), and transcatheter implantation of aortic valves (12–17). However, studies on the relationship between off-pump CABG-associated AKI and the inflammatory response are lacking.

Off-pump CABG-associated AKI also significantly increases the risk of renal replacement therapy and death in patients (18–20). The risk factors for the development of AKI behind off-pump CABG are not well understood. We investigated whether the ratios of cell types in peripheral blood could be predictors of off-pump CABG-associated AKI. We look forward to providing guidance for the early recognition and treatment of off-pump CABG-associated AKI.

## Materials and methods

### Ethical approval of the study protocol

This study protocol was approved by the Medical Ethics Committee of Qilu Hospital of Shandong University (Jinan, China). Written informed consent was waived and all patient information was stored anonymously.

### Study design

We retrospectively reviewed patients undergoing off-pump CABG from January 2021 to January 2022 at the Department of Cardiovascular Surgery within Qilu Hospital of Shandong University.

### Exclusion criteria

Patients were excluded if: (i) they needed intraoperative CPB; (ii) they had preoperative severe chronic kidney disease necessitating RRT; (iii) their postoperative serum creatinine (SCr) data were incomplete.

### Surgical procedures

Off-pump CABG was undertaken in patients with severe coronary artery disease [left main disease, three-vessel disease, combined with diabetes mellitus (DM)] or failed stenting. After the induction of general anesthesia with endotracheal intubation, a median sternal incision was made. The left internal mammary artery and great saphenous vein were freed as bridge vessels simultaneously. After heparinization, the anastomotic site was secured using a stabilizer. The anastomosis was undertaken with an intra-coronary shunt and deep pericardial suture. The operating surgeon measured the flow of vein grafts after the anastomosis to ensure the patency of grafted vascular bridges.

### Definition

We selected the most recent SCr value before the surgical procedure as the baseline level. We applied the Chronic Kidney Disease Epidemiology Collaboration equation to obtain the estimated glomerular filtration rate (eGFR) (21). The diagnosis and staging of AKI followed the criteria of the Kidney Disease: Improving Global Outcome (KDIGO) guideline (22) (Table 1).

**Table 1:** Stage of off-pump CABG-associated AKI following KDIGO criteria.

Stage	Serum creatinine	Urine volume
I	Increase ≥0.3 mg/dl (≥26.5 μmol/L) within 48 h or increase to 1.5–1.9-times baseline levels	<0.5 ml/kg/h for 6–12 h
II	Increase to 2.0–2.9-times baseline levels	<0.5 ml/kg/h for ≥12 h
III	Increase to ≥4.0 mg/dl (≥353 μmol/L) or increase to ≥3-times baseline levels or RRT initiation	<0.3 ml/kg/h for ≥24 h or anuria

AKI, acute kidney injury; KDIGO, Kidney Disease: Improving Global Outcome; RRT, renal replacement therapy.

We calculated the neutrophil:lymphocyte ratio (NLR), monocyte:lymphocyte ratio (MLR), and platelet:lymphocyte ratio (PLR) as biomarkers associated with the inflammatory response.

### Data collection

We documented the perioperative variables of patients. These were: age, gender, body mass index (BMI), tobacco smoking, hypertension, DM, hyperlipemia, history of cerebral diseases, kidney disease without RRT, chronic obstructive pulmonary disease (COPD), percutaneous coronary intervention (PCI), New York Heart Association (NYHA) functional classification, preoperative left ventricular ejection fraction (LVEF), preoperative peripheral blood counts, preoperative blood biochemistry, preoperative renal function, intraoperative erythrocyte transfusion, intraoperative urine volume, intraoperative fluid replacement, central venous pressure (CVP) and mean arterial pressure (MAP) at intensive care unit (ICU) admission, use of an intra-aortic balloon pump (IABP), low cardiac output syndrome (LCOS), RRT, application of vasoactive agents, duration of mechanical ventilation, peripheral blood counts on postoperative day (POD)1, postoperative renal function, postoperative erythrocyte transfusion, complications, duration of hospital stay, duration of ICU stay, death.

### Statistical analyses

We used SPSS 25.0 (IBM, Armonk, NY, United States) for statistical analyses. Measurement data were tested to see if they had a normal distribution. Variables with a normal distribution are expressed as the mean ± SD and were analyzed by the Student's *t*-test. Variables with a non-normal distribution are expressed as medians and quartiles and were analyzed by the Mann–Whitney *U*-test. Categorical data are expressed as frequencies and percentages and were compared by the chi-square test or Fisher's exact test. *P* < 0.05 (two-sided) was considered significant. Multivariate analysis incorporated variables with significant differences in univariate analysis. The results of multivariate logistic regression analysis are expressed as odds ratio (OR) and 95% confidence interval (CI). A receiver operating characteristic (ROC) curve and Hosmer–Lemeshow goodness of fit test were applied to evaluate the ability of predictive models. The maximum value of the Youden index was used to determine the cutoff value.

## Results

### Characteristics of the study cohort

**Figure 1:**
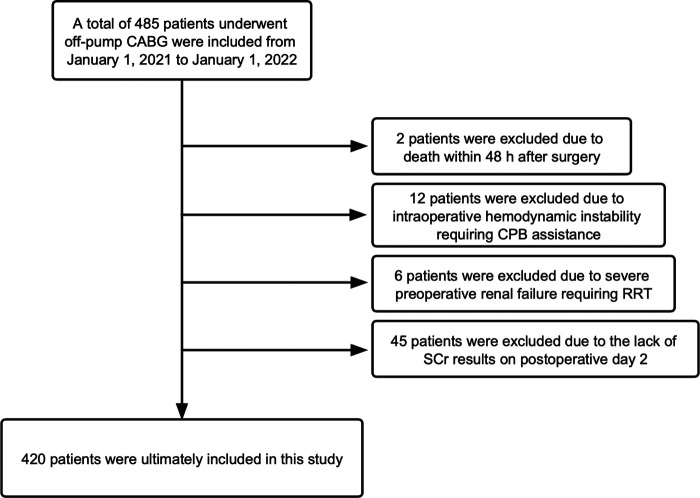
Flowchart of our study.

From January 1, 2021 to January 1, 2022, 485 patients underwent off-pump CABG in the Department of Cardiovascular Surgery within Qilu Hospital of Shandong University. We excluded 65 patients according to our exclusion criteria (Figure 1). Finally, the data of 420 patients were analyzed and their baseline characteristics are shown in Table 2.

**Table 2:** Characteristics of the study population.

Variable	All patients (*n* = 420)	Non-AKI (*n* = 332)	AKI (*n* = 88)	*P*
**Preoperative**				
Age (years)	65 (58, 69)	64 (58, 68)	67 (62, 72)	<0.01
Age ≥65	216 (51.4%)	159 (47.9%)	57 (64.8%)	<0.01
55 < age < 65	136 (32.4%)	116 (34.9%)	20 (22.7%)	0.029
Age ≤55	68 (16.2%)	57 (17.2%)	11 (12.5%)	0.290
Female	137 (32.6%)	100 (30.1%)	37 (42.0%)	0.034
BMI (kg/m^2^)	25.1 (23.2, 27.3)	25.2 (23.2, 27.3)	24.5 (22.8, 26.6)	0.171
Tobacco smoking	187 (44.5%)	151 (45.5%)	36 (40.9%)	0.443
Hypertension	257 (61.2%)	198 (59.6%)	59 (67.0%)	0.205
DM	174 (41.4%)	129 (38.9%)	45 (51.1%)	0.038
History of cerebral diseases	82 (19.5%)	63 (19.0%)	19 (21.6%)	0.582
Kidney disease without RRT	6 (1.4%)	2 (0.6%)	4 (4.5%)	<0.01
COPD	8 (1.9%)	6 (1.8%)	2 (2.3%)	0.776
PCI	50 (11.9%)	39 (11.7%)	11 (12.5%)	0.846
NYHA grade	—	—	—	<0.01
NYHA grade >2	152 (36.2%)	107 (32.2%)	45 (51.1%)	<0.01
NYHA grade ≤2	268 (63.8%)	225 (67.8%)	43 (48.9%)	<0.01
Hemoglobin (g/L)	137 (126, 147)	138 (127, 146)	133 (118, 148)	0.016
HCT (%)	41.10 (37.90, 43.50)	41.20 (38.45, 43.50)	39.10 (36.30, 43.35)	<0.01
Albumin (g/L)	42.30 (40.10, 44.30)	42.40 (40.40, 44.35)	41.80 (38.85, 44.05)	0.055
LDL (mmol/L)	1.97 (1.58, 2.49)	1.98 (1.57, 2.49)	1.97 (1.58, 2.49)	0.796
HDL (mmol/L)	0.99 (0.85, 1.13)	1.00 (0.85, 1.14)	0.96 (0.83, 1.09)	0.174
TG (mmol/L)	1.28 (0.96, 1.72)	1.24 (0.93, 1.69)	1.41 (1.08, 1.95)	0.017
Cys-C (mg/L)	1.01 (0.89, 1.15)	0.99 (0.88, 1.10)	1.14 (0.98, 1.37)	<0.01
BUN (mmol/L)	5.50 (4.52, 6.55)	5.40 (4.50, 6.40)	6.00 (5.00, 7.15)	<0.01
SCr (μmol/L)	76.0 (64.0, 86.0)	74.5 (64.0, 85.0)	82.5 (65.0, 97.0)	<0.01
eGFR (ml/min/1.73 m^2^)	93 (81, 101)	95 (85, 102)	87 (67, 97)	<0.01
eGFR ≥90	252 (60.0%)	215 (64.8%)	37 (42%)	<0.01
60 < eGFR < 90	144 (34.3%)	109 (32.8%)	35 (39.8%)	0.223
eGFR ≤60	24 (5.7%)	8 (2.4%)	16 (18.2%)	<0.01
LVEF (%)	60 (51, 65)	60 (53, 65)	57 (43, 62)	<0.01
LVEF ≥60	212 (50.5%)	177 (53.3%)	35 (39.8%)	0.024
50 < LVEF < 60	106 (25.2%)	84 (25.3%)	22 (25.0%)	0.954
LVEF ≤50	102 (24.3%)	71 (21.4%)	31 (35.2%)	<0.01
**Intraoperative**				
Emergency surgery	33 (7.9%)	23 (6.9%)	10 (11.4%)	0.169
Operation time (min)	270 (240, 300)	265 (240, 295)	273 (250, 295)	0.074
Erythrocyte transfusion (U)	0 (0, 2)	0 (0, 2)	1 (0, 4)	<0.01
Urine volume (ml)	700 (500, 1,000)	750 (500, 1,000)	700 (450, 1,000)	0.917
Fluid replacement (ml)	2,700 (2,500, 3,000)	2,700 (2,500, 3,000)	2,700 (2,500, 3,500)	0.189
**Postoperative**				
CVP (cmH_2_O)	8 (6, 10)	8 (6, 10)	8 (7, 11)	0.223
MAP (mmHg)	88 (76, 98)	88 (78, 98)	84 (72, 99)	0.259
Medicine application	160 (38.1%)	108 (32.5%)	52 (59.1%)	<0.01
Erythrocyte transfusion (U)	0 (0, 2)	0 (0, 2)	2 (0, 4)	<0.01
Mechanical ventilation (min)	780 (541, 1,140)	720 (513, 1,029)	1,200 (792, 3,390)	<0.01
LCOS	43 (10.2%)	14 (4.2%)	29 (33.0%)	<0.01
IABP	41 (9.8%)	14 (4.2%)	27 (30.7%)	<0.01
RRT	7 (1.7%)	0	7 (8.0%)	<0.01
Duration of hospital stay (day)	12 (10, 14)	12 (10, 14)	14 (12, 19)	<0.01
Duration of ICU stay (day)	3 (2, 4)	2 (2, 3)	4 (3, 7)	<0.01
28-day mortality	10 (2.4%)	1 (0.3%)	9 (10.2%)	<0.01

AKI, acute kidney injury; BMI, body mass index; BUN, blood urea nitrogen; COPD, chronic obstructive pulmonary disease; CVP, central venous pressure; Cys-C, cystatin C; DM, diabetes mellitus; eGFR, estimated glomerular filtration rate; HCT, hematocrit; HDL, high density lipoprotein; IABP, intra-aortic balloon pump; ICU, intensive care unit; LCOS, low cardiac output syndrome; LDL, low density lipoprotein; LVEF, left ventricular ejection fraction; MAP, mean arterial pressure; NYHA, New York Heart Association; PCI, percutaneous coronary intervention; RRT, renal replacement therapy; SCr, Serum creatinine; TG, triglyceride.

Eighty-eight patients (20.95%) developed AKI after off-pump CABG (67 patients with stage-I, 7 patients with stage-II, and 14 patients with stage-III AKI). Sixteen patients were diagnosed with AKI on POD1. The peak value of SCr occurred on POD2. Patients in the AKI group had a longer stay in the ICU and hospital. Seven patients received RRT after off-pump CABG, and 10 patients died within 28 days after CABG.

### Inflammation-related biomarkers and off-pump CABG-associated AKI

We measured the levels of inflammation-related biomarkers before and on POD1 (Table 3). The counts for leukocytes, neutrophils, monocytes, and the levels of procalcitonin (PCT), which are correlated positively with inflammation, increased significantly on POD1. The lymphocyte count and platelet count, which are correlated negatively with the inflammatory response, decreased significantly on POD1. These results demonstrated that patients undergoing off-pump CABG experienced a dramatic inflammatory response. Moreover, there were significant differences in the ratios of cell types in peripheral blood on POD1 between patients in the AKI group and non-AKI group (*P* < 0.01), which implied a more pronounced inﬂammatory response in the AKI group. Patients in the AKI group had significantly higher levels of interleukin (IL)-6 than patients in the non-AKI group on POD1 (Supplementary Table S1 and Supplementary Figure S1).

**Table 3:** Inflammation-related biomarkers in the non-AKI and AKI group.

Variables	All patients (*n* = 420)	Non-AKI (*n* = 332)	AKI (*n* = 88)	*P*
**Preoperative inflammation-related biomarkers**				
WBC (10^9^/L)	6.37 (5.26, 7.45)	6.33 (5.27, 7.49)	6.50 (5.14, 7.33)	0.685
NEU (10^9^/L)	3.96 (3.08, 4.86)	3.93 (3.05, 4.82)	4.19 (3.17, 4.94)	0.264
LYM (10^9^/L)	1.60 (1.31, 1.96)	1.60 (1.32, 1.97)	1.53 (1.27, 1.83)	0.160
MON (10^9^/L)	0.46 (0.37, 0.57)	0.46 (0.37, 0.57)	0.45 (0.39, 0.57)	0.841
PLT (10^9^/L)	224 (186, 265)	224 (186, 263)	230 (197, 284)	0.347
NLR	2.47 (1.84, 3.15)	2.40 (1.80, 3.07)	2.64 (2.13, 3.25)	0.034
MLR	0.28 (0.22, 0.37)	0.28 (0.22, 0.37)	0.30 (0.23, 0.37)	0.200
PLR	138 (111, 173)	135 (110, 172)	145 (123, 179)	0.058
LDH (U/L)	210 (187, 241)	211 (188, 240)	210 (185, 247)	0.752
**Inflammation-related biomarkers on POD1**				
WBC (10^9^/L)	12.22 ± 3.89	12.20 ± 3.90	12.25 ± 3.88	0.915
NEU (10^9^/L)	10.78 ± 3.54	10.70 ± 3.52	11.09 ± 3.58	0.359
LYM (10^9^/L)	0.56 (0.41, 0.73)	0.60 (0.44, 0.78)	0.41 (0.31, 0.50)	<0.01
MON (10^9^/L)	0.72 (0.51, 0.98)	0.74 (0.52, 0.99)	0.68 (0.48, 0.92)	0.102
PLT (10^9^/L)	166 (133, 206)	166 (134, 204)	166 (129, 219)	0.908
NLR	19.13 (13.53, 26.34)	17.18 (12.63, 23.62)	26.40 (21.04, 33.93)	<0.01
MLR	1.30 (0.92, 1.74)	1.24 (0.89, 1.61)	1.74 (1.22, 2.44)	<0.01
PLR	302 (212, 415)	271 (203, 393)	382 (300, 526)	<0.01
PCT (ng/ml)	1.31 (0.49, 3.16)	1.13 (0.44, 2.59)	2.32 (0.68, 5.61)	<0.01
LDH (U/L)	211 (175, 259)	209 (173, 254)	219 (181, 302)	0.071

AKI, acute kidney injury; LDH, lactate dehydrogenase; LYM, lymphocyte; MLR, monocyte:lymphocyte ratio; MON, monocyte; NEU, neutrophil; NLR, neutrophil:lymphocyte ratio; PCT, procalcitonin; PLR, platelet:lymphocyte ratio; PLT, platelet; POD, postoperative day; WBC, white blood cell.

In addition, we found that ten of the patients who were diagnosed with AKI on POD1 developed more severe AKI in the following days. We divided them into the deteriorate and stable group based on their development of AKI. Patients in the deteriorate group had higher NLR on POD1 than those in the stable group, which indicated that NLR on POD1 may be used as predictors for more severe AKI in these patients (Supplementary Table S2 and Supplementary Figure S2).

**Figure 2:**
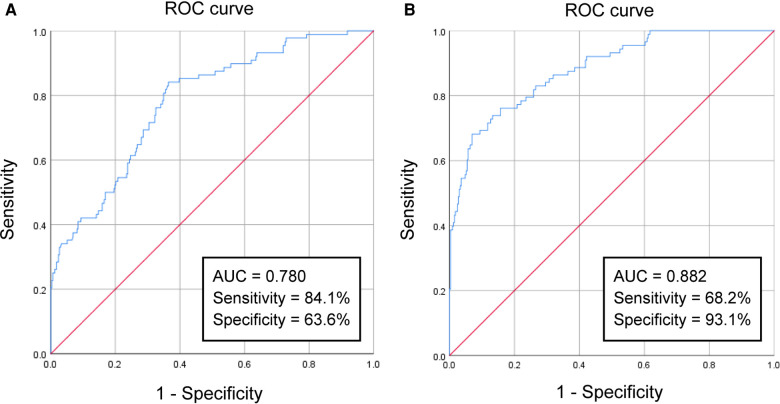
The results of ROC-curve analysis: (**A**) the ROC curve of the NLR for predicting off-pump CABG-associated AKI. (**B**) The ROC curve of the predictive model.

### Independent risk factors of off-pump CABG-associated AKI

The results of univariate analysis exhibited that there were statistical differences between AKI and no-AKI groups regarding older than 65, female gender, DM, kidney disease without RRT, NYHA score greater than 2, hemoglobin, hematocrit, triglyceride, preoperative cystatin C, preoperative blood urea nitrogen, preoperative SCr, eGFR below 60%, LVEF below 50%, application of vasoactive agents, erythrocyte transfusion, duration of mechanical ventilation, LCOS, use of an IABP, preoperative NLR, NLR on POD1, MLR on POD1, PLR on POD1, PCT on POD1 (Table 2 and Table 3).

We included variables described above into a multivariate logistics regression model. Being female (OR = 3.200, 95%CI = 1.118–9.115), total erythrocyte transfusion (1.157, 1.019–1.31), NLR on POD1 (1.149, 1.071–1.232), PCT level on POD1 (1.061, 1.021–1.102), and duration of mechanical ventilation (1.027, 1.009–1.045) were independent risk factors of off-pump CABG-associated AKI (Table 4).

**Table 4:** Independent risk factors of off-pump CABG-associated AKI.

Variables	*B*	*P*	OR	95%CI
Female	1.163	0.030	3.200	1.118–9.155
Total erythrocyte transfusion	0.145	0.024	1.157	1.019–1.313
NLR on POD1	0.139	<0.01	1.149	1.071–1.232
PCT on POD1	0.059	<0.01	1.061	1.021–1.102
Mechanical ventilation	0.026	<0.01	1.027	1.009–1.045

AKI, acute kidney injury; NLR, neutrophil:lymphocyte ratio; PCT, procalcitonin; POD, postoperative day.

### Predictive model of off-pump CABG-associated AKI

We used ROC-curve analysis to calculate the predictive ability of the NLR on POD1. The area under the ROC curve (AUC) of the NLR for predicting off-pump CABG-associated AKI was 0.780 (Figure 2A). The sensitivity was 84.1% and the specificity was 63.6%. When the Youden index reached a maximum, the cutoff value of the NLR was 20.07. Next, we included all the independent risk factors obtained by multivariate analysis into a predictive model for ROC-curve analysis. The AUC of the new predictive model was 0.882 (Figure 2B), which exhibited a better predictive ability. The sensitivity was 68.2% and the specificity was 93.1%. And the *P* value of Hosmer–Lemeshow goodness of fit test equals 0.074 (*P* > 0.05).

### Correlation between the NLR and postoperative complications

We divided patients into a high-NLR group and low-NLR group according to the cutoff value (20.07) of the NLR on POD1. A high NLR on POD1 was closely associated with more severe AKI, pulmonary infection, hydrothorax, severe respiratory failure, and malignant arrhythmia (Table 5). Moreover, postoperative 28-day mortality was significantly higher in patients with a high NLR than in those with a low NLR (*P* < 0.05). These results demonstrated that patients with a high NLR on POD1 had poor short-term outcomes.

**Table 5:** Correlation between the NLR and postoperative complications.

Complications	All patients (*n* = 420)	Low-NLR group (*n* = 225)	High-NLR group (*n* = 195)	*P*
Stage-I AKI	67 (16.0%)	13 (5.8%)	54 (27.7%)	<0.01
Stage-II AKI	7 (1.7%)	0	7 (3.6%)	<0.01
Stage-III AKI	14 (3.3%)	1 (0.4%)	13 (6.7%)	<0.01
Cerebral infarction	14 (3.3%)	5 (2.2%)	9 (4.6%)	0.173
Pulmonary infection	130 (31.0%)	59 (26.2%)	71 (36.4%)	0.024
Incision infection	5 (1.2%)	1 (0.4%)	4 (2.1%)	0.130
Multiple operations	6 (1.4%)	1 (0.4%)	5 (2.6%)	0.068
Hydrothorax	110 (26.2%)	44 (19.6%)	66 (33.8%)	<0.01
Severe respiratory failure	36 (8.6%)	11 (4.9%)	25 (12.8%)	<0.01
Atrial fibrillation	107 (25.5%)	52 (23.1%)	55 (28.2%)	0.232
Malignant arrhythmia	16 (3.8%)	2 (0.9%)	14 (7.2%)	<0.01
28-day mortality	10 (2.4%)	2 (0.9%)	8 (4.1%)	0.031

AKI, acute kidney injury; NLR, neutrophil:lymphocyte ratio.

## Discussion

Ischemic AKI is the most prevalent type of CSA-AKI. According to the change in the GFR, the development of ischemic AKI can be divided into four phases: initiation, extension, maintenance, and recovery (23). In the initiation phase, ischemia-induced damage to tubular epithelial cells and endothelial cells leads to the release of chemokines and cytokines that activate inflammatory cascades (24). The inflammatory response aggravates tubular-cell injury in the extension phase, which leads to a continued reduction in the GFR. Significant infiltration of inflammatory cells in the renal outer medulla occurs as early as 24 h after ischemia (25), and leukocytes may appear as early as 2 h after ischemia (26). Therefore, early identification and interrupting the amplification of the inflammatory response in the extension phase is very important.

The SCr level peaked on POD2 and the infiltration of inflammatory cells would appear early, so we chose the cell ratios in peripheral blood on POD1 as biomarkers. Our study found that the NLR on POD1 was a reliable biomarker for the early prediction of off-pump CABG-associated AKI (AUC = 0.780, cutoff = 20.07). The NLR was derived from the hematological observation that the neutrophil count is associated positively with cardiovascular events and the lymphocyte count is associated negatively with cardiovascular events (27, 28). Neutrophil activation is an important sign of acute inflammatory response, and lymphopenia is a marker of poor health condition and physiological stress. Compared with C-reactive protein, IL-6, and other inflammation-specific biomarkers, the NLR can be obtained more readily and in an inexpensive manner, so it has attracted the attention of researchers. A meta-analysis involving five randomized clinical trials with large cohorts showed that the NLR at baseline independently predicted the risk of cardiovascular events and all-cause mortality in patients (29). Kim and colleagues revealed a high NLR on POD1 to be closely associated with an increased risk of CSA-AKI and 1-year mortality (30).

The high NLR level on POD1 may indicate the early inflammatory response after off-pump CABG, which is one of the most important mechanisms of AKI. Many animal experiments have proved the important role of inflammatory response in the development of AKI. Kelly and colleagues found that anti-intercellular adhesion molecule-1 therapy prevented ischemic AKI in mice *via* neutrophil-dependent pathway (31, 32). Rabb and coworkers confirmed that the adhesion molecules CD11 and CD18 on the surface of leukocytes play an important role in ischemic AKI in rats (33). In addition, selectin ligand inhibitors also successfully attenuated renal ischemia-reperfusion injury in rat and pig models (34, 35). Besides the adhesion molecules described above, chemokines, proinflammatory cytokines, reactive oxygen species, C-reactive protein and danger-associated molecular patterns also participate in the inflammatory response leading to unacceptable AKI.

CPB during cardiac surgery activates the immune system significantly: a large number of cytokines and chemokines are released, which increases the risk of AKI (36). Parlar and coworkers found that the postoperative NLR was an independent predictor of AKI after on-pump CABG (37). However, we found that the leukocyte count, PCT level, and IL-6 level were also significantly higher in patients after off-pump CABG, which reflected a marked inflammatory response. Studies correlating inflammation with off-pump CABG-associated AKI are lacking. Our findings demonstrated this association and revealed the predictive value of the NLR on POD1.

We also discovered four other independent risk factors of off-pump CABG-associated AKI according to multivariate analysis: gender, total erythrocyte transfusion, PCT level on POD1, and duration of mechanical ventilation. In our study, female patients accounted for 32.6% of the study cohort, but the prevalence of AKI was much higher than that in male patients (*P* < 0.05). After adjustment for other variables, being female remained an independent risk factor of off-pump CABG-associated AKI. This observation is consistent with findings in some studies (38, 39) but not in other studies (40, 41).

The free hemoglobin and free iron released from an erythrocyte transfusion would cause oxidative stress, which aggravates inflammation and ischemia–reperfusion injury in the kidney (42, 43). Therefore, many researchers expect to reduce the occurrence of CSA-AKI by restricting erythrocyte transfusion (44, 45). The association between mechanical ventilation and AKI was documented first by Drury and coworkers in 1947. They found that continuous-pressure ventilation caused a decline in renal function (46). Afterwards, Kuiperet and colleagues proposed that mechanical ventilation may affect renal function due to hemodynamic alterations and ventilator-induced lung injury which activates a systemic inflammatory response (47). We also found a correlation between higher levels of inflammatory biomarkers on POD1 and pulmonary complications.

PCT is a commonly used indicator for the diagnosis of sepsis in the ICU. The PCT level can be used to assess bacterial infection in the body. Studies of PCT and AKI have focused mainly on patients with sepsis, with fewer studies concentrating on patients undergoing cardiac surgery. Heredia-Rodríguez and colleagues enrolled patients with a systemic inflammatory response or sepsis after cardiac surgery. They showed that the PCT level was significantly higher in patients with CSA-AKI (48). Our results also confirmed this correlation, and we found that a high PCT level on POD1 was an independent risk factor of off-pump CABG-associated AKI. We hypothesize that patients on POD1 did not develop a bacterial infection, but the high PCT level may suggest an inflammatory reaction occurring *in vivo* and a higher risk of infection.

We included the independent risk factors stated above into a prediction model and obtained a high predictive ability (AUC = 0.882). Our study provides a predictive biomarker and predictive model for the early recognition and timely intervention of off-pump CABG-associated AKI. We aim to improve the prognosis of patients undergoing off-pump CABG.

Our study had three main limitations. First, this was a single-center retrospective study. The predictors obtained and prediction model created must undergo external validation. Second, we assessed only the short-term outcomes of patients after off-pump CABG. Third, the predictive value of NLR on POD1 was attenuated in patients who have had AKI on POD1. In the future, we will dedicate ourselves to improving the intraoperative management of off-pump CABG and carrying out a prospective study that documents the prevalence of postoperative AKI in patients undergoing different treatments.

## Conclusion

We demonstrated the NLR on POD1 to be a reliable biomarker for predicting off-pump CABG-associated AKI. Also, we constructed a prediction model that may contribute to the early recognition and management of off-pump CABG-associated AKI.

## Data Availability

The original contributions presented in the study are included in the article/**Supplementary Material**, further inquiries can be directed to the corresponding author/s.
