# Supplementary material for: Predictive role of the neutrophil: lymphocyte ratio in acute kidney injury associated with off-pump coronary artery bypass grafting
Source: Front Surg. 2022 Nov 8;9:1047050. doi: 10.3389/fsurg.2022.1047050 (PMC9679147; doi:10.3389/fsurg.2022.1047050)
Supplement: Supplementary file 1 [file Datasheet1.docx]

Supplementary Material

# Supplementary Table 1 The IL-6 level in the non-AKI and AKI group on POD1.

|  | ALL patients  (n = 208) | Non-AKI group  (n = 165) | AKI group  (n = 43) | P |
| --- | --- | --- | --- | --- |
| IL-6 on POD1 | 255.85 (150.40, 619.70) | 225.10 (143.50, 520.90) | 447.30 (263.55, 1124.50) | < 0.01 |

The IL-6 levels on POD1 in 212 patients were lacking.

# Supplementary Table 2 The NLR on POD1 in patients who developed AKI on POD1.

| Variables | All patients  (n = 16) | Stable group  (n = 6) | Deteriorate group  (n = 10) | P |
| --- | --- | --- | --- | --- |
| NEU on POD1 (10^9^/L) | 11.39 ± 3.74 | 9.74 ± 3.79 | 12.38 ± 3.52 | 0.180 |
| LYM on POD1 (10^9^/L) | 0.46 ± 0.12 | 0.52 ± 0.12 | 0.42 ± 0.11 | 0.128 |
| NLR on POD1 | 24.89 (21.33, 30.00) | 18.73 (15.09, 25.39) | 28.83 (24.38, 30.13) | 0.023 |

# Abbreviations: AKI, acute kidney injury; LYM, lymphocyte; NEU, neutrophil; NLR, neutrophil:lymphocyte ratio; POD, postoperative day.

# Supplementary Figure 1

**
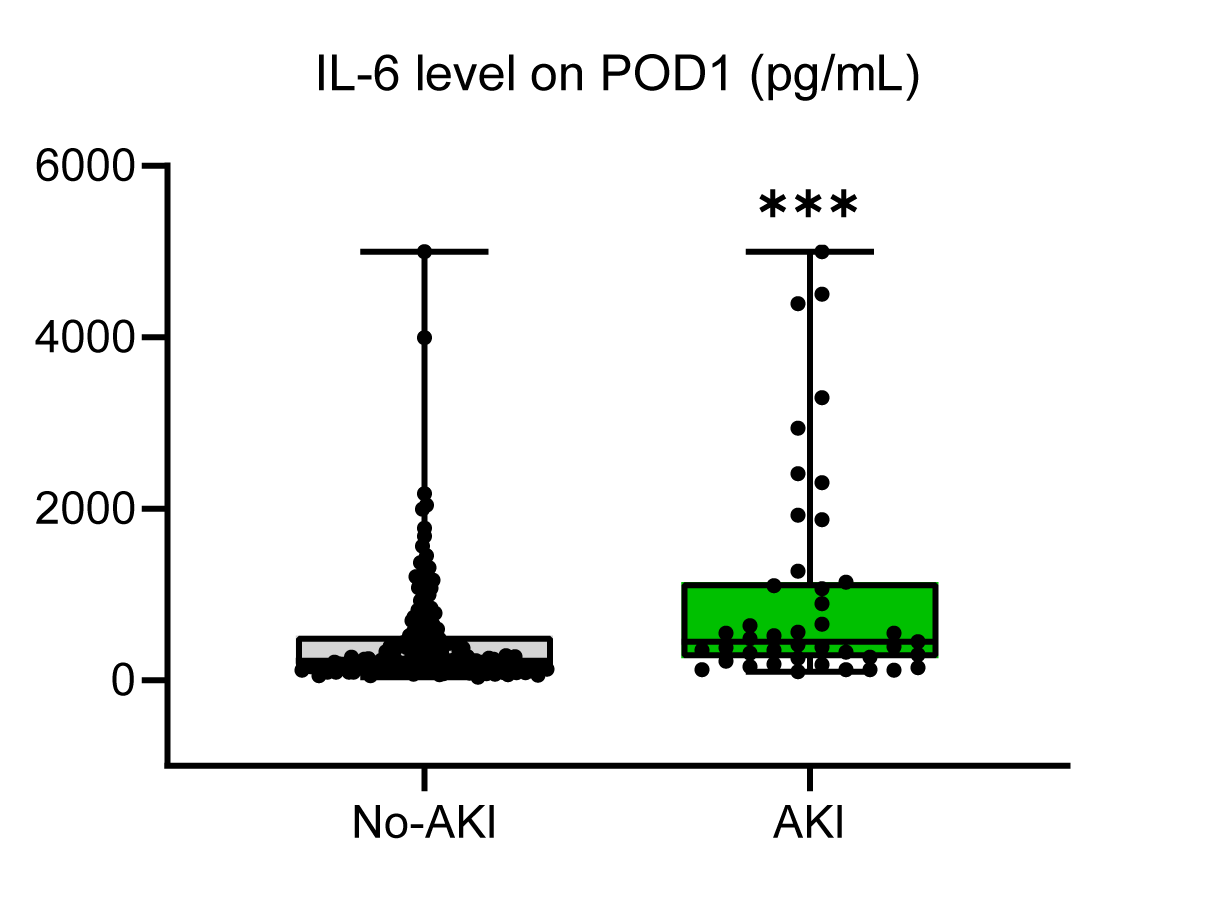
**

# **Supplementary Figure 1.** The IL-6 level in the non-AKI and AKI group on POD1.

# Supplementary Figure 2

**
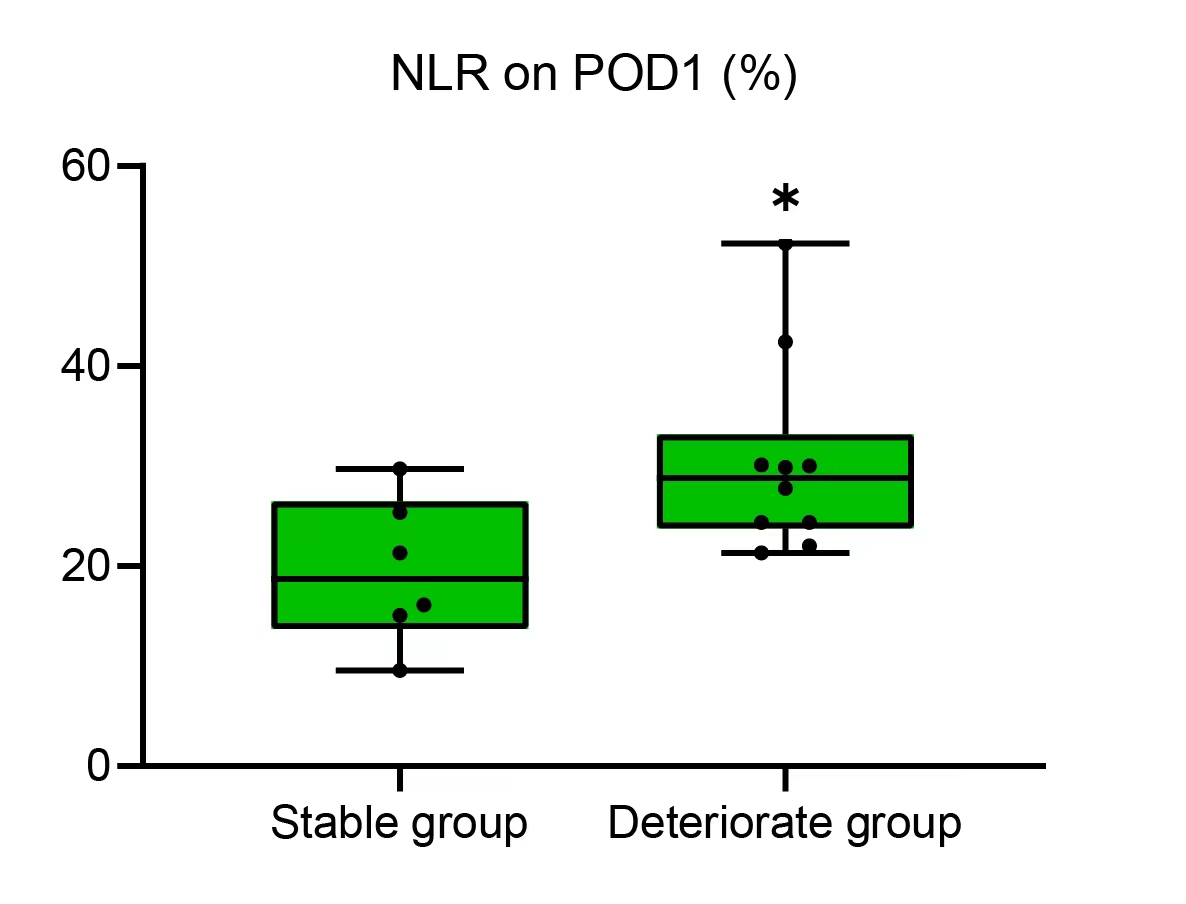
**

**Supplementary Figure 2.** The NLR on POD1 in patients who developed AKI on POD1.
